# Characterization of Myrrh Extract Effect on Polylactide and Polypropylene Melt Spun Multifilament Yarn Structure and Properties

**DOI:** 10.3390/ma17235843

**Published:** 2024-11-28

**Authors:** Evaldas Bolskis, Egidijus Griškonis, Mindaugas Marksa, Lina Ragelienė, Erika Adomavičiūtė

**Affiliations:** 1 Faculty of Mechanical Engineering and Design, Kaunas University of Technology, Studentu St. 56, 51424 Kaunas, Lithuania; erika.adomaviciute@ktu.lt; 2 Faculty of Chemical Technology, Kaunas University of Technology, Radvilenu Rd. 19, 50254 Kaunas, Lithuania; egidijus.griskonis@ktu.lt; 3 Department of Analytical and Toxicological Chemistry, Lithuanian University of Health Sciences, Sukileliu Avenue 13, 50162 Kaunas, Lithuania; mindaugas.marksa@lsmu.lt; 4 Faculty of Natural Sciences, Vytautas Magnus University, Universiteto St. 10, Akademija, 53362 Kaunas, Lithuania; lina.rageliene@vdu.lt

**Keywords:** PLA, melt spinning, myrrh, multifilament yarns, antibacterial activity

## Abstract

Myrrh has unique medicinal properties: it is an anti-inflammatory, antifungal, and antibacterial material. The aim of this study was to assess the influence of ethanolic myrrh extract on the production and properties of modified PP and PLA melt spun yarns. In this work, multifilament yarns of polylactide (PLA) and polypropylene (PP) containing 10 wt% myrrh resin at different melt-spinning drawing ratios (DRs) were prepared. The results of scanning electron microscopy revealed that the multifilament yarns from polymers covered by myrrh resin extract had a smooth surface without cracks or visible myrrh derivatives. The influence of myrrh resin on the mechanical properties of PP and PLA multifilament yarns was analyzed, and it was found that the presence of myrrh (PP/M, PLA/M) increased tenacity (cN/tex) and decreased the tensile strain (%) of melt spun yarns obtained at different draw ratios (DRs). During optical analysis, it was found that the absorbance of yarns increased in the entire UV region of the spectra, which was most likely determined by the presence of myrrh. The degree of crystallinity and the wetting angle of PP/M and PLA/M multifilament yarns increased compared with the pure PLA and PP multifilament yarns. This study concludes that the presence of myrrh derivatives influences PLA yarns degradation rate and antibacterial effects against Gram-positive bacteria.

## 1. Introduction

Manmade fibers can be produced by various methods. Conventional chemical textile fibers are manufactured through wet, dry, or melt spinning processes. The melt-spinning method is easy to use, adaptable to different systems and conditions, and suitable for large-scale productions. It is an inexpensive and quick processing method that requires no auxiliary agents, such as solvents [1,2,3].

Many polymers can be used in the melt-spinning process, such as polyesters (poly(ethylene terephthalate) (PET), poly(butylene terephthalate) (PBT)) [4,5,6], polyurethanes, polyolefins (polypropylene (PP), high-density polyethylene (HDPE), low-density polyethylene (LDPE)) [7,8,9], polyamides (PA 6, PA 5) [8,10,11], and biopolymers (polylactic acid (PLA), polycaprolactone (PCL), poly(glycolic acid) (PGA), poly(butylene adipate-co-terephthalate) (PBAT), etc.) [12,13,14,15].

PP is a nonbiodegradable, thermoplastic polymer with good processing capability, weldability, and mechanical properties. Due to the good compatibility of PP with various modifiers, fillers, and reinforcing fillers, it has become a popular polymer with a wide range of applications [16]. The great thermal stability of PP makes it suitable for medical applications where sterilization at relatively high temperatures is required. PP sutures and hernia meshes have good resistance, stability, and low-tissue reaction [17,18,19]. PP nanocomposites are one of the rarely explored thermoplastic hybrids in fiber technology. Nanoparticles such as silver (Ag), titanium dioxide (TiO_2_), silica (SiO_2_), zinc oxide (ZnO), and copper oxide (CuO) have been incorporated into PP [11,20,21,22,23,24]. Also, by using PP with stearic acid [25], paraffin microcapsules [26], and carbon nanotubes [27], composite fibers have been successfully melt spun.

PLA has been proven to be an ideal substitute for petroleum-derived polymers, and biodegradable polymers have good biocompatibility and processability in melt-spinning processes. The raw materials for PLA preparation come from renewable crops, such as corn, sugarcane, and cassava [28]. PLA with carbon nanotubes [29], cellulose nano whiskers [30], pine rosin [31], silver nanoparticles (AgNPs) [32], and Ag/PVP [33] have been successfully melt spun to yarns. M. Kanerva and coauthors [31] formed PLA melt spun yarns with 20 wt% pine rosin using bioreagent-type copolymer surfactant (PF) F-127 by Pluronic; without surfactant, only 10 wt% rosin could be used in the formation of PLA yarns.

Modified multifilament yarns can be used as antibacterial materials. Two types of additional species can be used in the formation of multifilament yarns: inorganic (nanoparticles or microparticles of metals and their oxides, minerals) and organic (flavonoids, alkaloids, etc.). With regard to the antibacterial properties of multifilament yarns, the most effective inorganic materials that have been used in antimicrobial applications in textiles are AgNPs, Ag/PVP, SiO_2_, TiO_2_, and ZnO [21,23,33,34]. Recently, more research has focused on the use of natural compounds for antibacterial properties. Natural compounds may be from plants, vegetables, fruits, or other natural resources that are rich in various bioactive compounds such as flavonoids, alkaloids, etc. [35]. These compounds demonstrate various biological effects, such as antibacterial, anti-inflammatory, or antioxidant effects. Their effect (biological activity) can be used in the medical field by inserting these materials into the medical textiles [36]. Natural compounds have already been added to polymer films, such as coffee, cacao, basil oil extract, oregano, geraniol, and cinnamon oil [37,38,39,40,41,42]. Melt spun yarns have been formed with colophony [31], natural pine/spruce rosin [8,31], and propolis [18]. One natural compound is myrrh. Myrrh can be defined as an oleogum resin produced by different species of Commiphora. It is composed of 3–4 wt% impurities, 7–17 wt% volatile oils, 25–40 wt% alcohol-soluble resins, and 57–61 wt% water-soluble gum [43]. Myrrh has unique medicinal properties: it can act as an anti-inflammatory, antifungal, astringent, analgesic, antiseptic, and diuretic agent [44,45,46]. In previous studies [42], it was shown that the best way to form PLA melt spun yarns is by using ethanolic myrrh extract instead of water-based myrrh extract.

The aim of this study was to assess the influence of ethanolic myrrh extract on the production of PP and PLA melt spun yarns, investigating the mechanical, thermal, optical, and surface-wetting properties. We analyzed the antibacterial properties of PLA yarns in order to prove the hypothesis that melt spun yarns with myrrh ethanolic extract may be suitable for medical purposes.

## 2. Materials and Methods

### 2.1. Materials

Melt spun multifilament yarns were produced from polypropylene (PP) H253 FF/3 granules (Sibur International GmbH, Vienna, Austria) and fiber-grade polylactic acid (PLA) 6202D granules (Nature Works, Blair, NE, USA) with an average molecular weight of 140 kDa. Myrrh resin was imported from India (Ekokolekcija, Vilnius, Lithuania). Ethanol (96 vol%) was used as a solvent for the production of myrrh resin extract.

### 2.2. Methodology of Preparation of Ethanolic Myrrh Extract

The myrrh resin particles were ground into fine powder before the extraction process. For the extraction of raw myrrh, 96% ethanol was used. The ethanolic extract of myrrh was prepared by heating it at 90 °C for 12 h in a round-bottomed flask equipped with an Allihn condenser. The flask was placed in a sand bath and stirred at 400 rpm using a magnetic stirrer (IKA RH, basic KT/C, Staufen, Atlanta, GA, USA). The concentration of the obtained ethanolic myrrh extract was 30%. The ethanolic myrrh extract was filtered through a Buchner funnel with Filtrak No. 389 filter paper to remove undissolved solids, such as sand and ground material [42].

### 2.3. Gas Chromatography–Mass Spectrometry Analysis of Ethanolic Myrrh Extract

The ethanolic myrrh extract was analyzed using gas chromatography–mass spectrometry (GC/MS) (SHIMADZU GC/MS-QP2010nc Ultra; Shimadzu Technologies, Kyoto, Japan) equipped with a Shimadzu AOC-5000 autoinjector (Shimadzu, Tokio, Japan). A capillary column RXi-5MS (30 m × 0.25 mm i.d. × 0.25 film thickness µm) (Restek, Bellefonte, PA, USA) was used for analysis. The injection volume was 1 µL, the split ratio was 1:5 (v:v), and the split injector temperature was 260 °C. Helium was used as the carrier gas with a flow rate of 1.22 mL min^−1^. The column temperature was initially set to 50 °C and held for 5 min, then increased to 200 °C at the rate of 2 °C min^−1^, and then to 315 °C at the rate of 15 °C min^−1^, where it was held for another 5 min. The temperature of the detector ion source and the interface were 200 °C and 280 °C, respectively. Mass spectra were acquired at an ionization voltage of 70 eV, a scan rate of 2500 *m*/*z* within the range of 29–500 *m*/*z*, and a scan time of 0.2 s.

### 2.4. Covering of Polymer Granules with Ethanolic Myrrh Extract

The polymer granules (PP and PLA) were covered with ethanolic myrrh resin solution by a spraying process. The polymer granules were sprayed with ethanolic myrrh resin extract, mixed with a glass rod in a Teflon dish, and dried at 80 °C for at least 60 min until the ethanol had evaporated. This procedure was repeated several times, until PP and PLA granules were loaded with 10 wt% of myrrh resin.

### 2.5. Melt Spinning of Multifilament Yarns

Four types of multifilament yarns were made: pure polymer PLA and PP yarns and yarns made from PP and PLA granules coated with ethanolic myrrh extract (PP/M and PLA/M, respectively). The yarns were obtained using a COLLIN^®^ CMF 100 (Collin GmbH, Maitenbeth, Germany) single-screw extruder [42]. All PLA granules were dried prior to extrusion. The water content of the granules was determined using an automated Karl Fischer titrator (756 KF coulometer equipped with a oven sample processor 774 (Metrohm, Herisau, Switzerland). The targeted water content levels were below 45 ppm. The temperature in all seven heating zones of the single-screw extruder (L/D ratio of 25:1) was set to 205 °C. The average extruder speed was set at 29 rpm. Circular spinnerets with 24 holes (diameter 0.45 mm) were used. The filaments were cooled using crossflow air quenching at a temperature of 14 °C. The temperature of the stretching rolls was 75 °C in all experiments. Multifilament yarns were formed by varying the speed of stretching rolls, as presented in Table 1.

### 2.6. Linear Density of Melt Spun Multifilament Yarns

Linear density of the yarns was measured according to the method described in our previous work [42]. For the tests, the PP, PLA, PP/M, and PLA/M multifilament yarns were conditioned for 24 h in the standard atmosphere according EN ISO 2060:1994 [47], i.e., at a relative humidity of (φ = 65% ± 4%) and temperature of 20 °C ± 2 °C. The specimens of 50 m in length were prepared by reeling skeins using a Zweigle L232 machine (Zweigle Textilprüfmaschinen GmbH & Co., Reutlingen, Germany), to measure the linear density of multifilament yarns. The mass of the skeins was determined under standard atmospheric conditions using laboratory scales (KERN EW150-3M; Kern & Sohn GmbH, Balingen, Germany).

### 2.7. Scanning Electron Microscopy (SEM) Analysis of Melt Spun Multifilament Yarns

Morphology and diameters of the melt spun multifilament yarns were determined using an SEM S-3400N scanning electron microscope (Hitachi, Tokyo, Japan (beam voltage: 3 kV, magnification: 50×, scale bar: 1 mm)). The diameters of the fibers were evaluated using SEM images and NIS-Elements D (Nikon Corporation, Tokyo, Japan). The average diameter of 140 microfibers was calculated using measurements obtained from SEM images.

### 2.8. Mechanical Properties of Melt Spun Multifilament Yarns

Mechanical properties (tenacity (cN/tex) and tensile strain (%)) of the PLA and PP multifilament yarns were determined according to the EN ISO 2062:2009 standard [48]. The experiments were conducted in a standard atmosphere at a temperature of 20 ± 2 °C and a relative humidity of 65 ± 4%. Universal testing equipment (Zwick/Roell; Zwick GmbH & Co. KG, Ulm, Germany) with the testXpert^®^ (version V11.02 software) operating programme was used. The length between the clamps was 100 mm for the PP and PP/M multifilament yarns and 250 mm for the PLA and PLA/M multifilament yarns. A stretching speed of 500 mm/min and a pretension of 0.5 cN/tex were used. The number of tensile tests was 35.

### 2.9. Optical Properties of Melt Spun Multifilament Yarns

The UV–Vis diffuse reflectance spectra of PP and PLA melt spun multifilament yarns were measured using a Lambda 35 UV/VIS spectrometer (Perkin-Elmer, Waltham, MA, USA) over a wavelength range of 200–800 nm. Diffuse reflectance measurements of PP3, PP/M3, PLA3, and PLA/M3 multifilament yarns were performed by wrapping multiple layers (no fewer than 10) around the microscope coverslip.

### 2.10. Thermal Behavior of Melt Spun Multifilament Yarns

Differential scanning calorimetry (DSC) was performed on the melt spun multifilament yarns using a Netzsch Polyma DSC 214 (NETZSCH-Gerätebau GmbH, Selb, Germany). The experiments were conducted on pure PP3, PLA3, PP/M3, and PLA/M3 multifilament yarns to obtain glass transition, melting, crystallization, and cold crystallization temperatures. Approximately 5 mg of each sample was weighed into separate aluminum pans. The heating and cooling scan rates were set to 10 °C/min^−1^ under a nitrogen atmosphere with a flow rate of 20 mL/min^−1^. The procedure was as follows: first, a heating scan at 10 °C/min from 15 °C up to 240 °C, then an isotherm at 240 °C for 3 min, followed by a cool down at 10 °C/min to 15 °C, and an isotherm at 15 °C temperature for 3 min. Then, a second heating scan with a ramp-up of 15 °C to 240 °C at 10 °C/min^−1^ was performed. The degrees of crystallinity of pure PLA and PLA saturated with resin samples were calculated using the following equation [49,50]:

(1) $$x_{c}\left(\%\right)=\frac{{\Delta H}_{m}-{\Delta H}_{c}}{{\lambda \Delta H}_{m1},o}\times 100\%$$

where ΔH_m_—melting enthalpy of PLA (J/g); ΔH_c_—cold crystallization enthalpy of PLA (J/g); λ—mass fraction of PLA in composite yarns; ΔH_m1_,o—PLA melting enthalpy of 100% crystal, of which the value was 93.6 J/g [51].

The degree of the crystallinity of PP and PP/M samples was calculated using the following equation [52]:

(2) $$x_{c}\left(\%\right)=\frac{{\Delta H}_{m}}{{\lambda \Delta H}_{m2},o}\times 100\%$$

where ΔH_m_—melting enthalpy of PP (J/g); λ—mass fraction of PP in composite yarns; ΔH_m2_,o—PP melting enthalpy of PP of 100% crystal, of which the value was 209 J/g [18].

### 2.11. Chemical Interactions Between Myrrh Resin and PP, PLA

The Raman scattering measurement was performed using a Raman microscope (inVia; Renishaw, Kingswood, UK). To record the spectra, an exciting wavelength (λ = 633 nm) was used that was provided by a helium neon laser. All peaks were obtained using a 50× lens. The excitation beam from a diode laser with a wavelength of 532 nm was focused on the sample using a 50× objective (NA = 0.75, Leica). The laser power at the sample surface was 0.15–0.3 mW. For all measurements, the integration time was 10 s for all measurements. The Raman Stokes signal was dispersed using a diffraction grating (2400 grooves/mm), and the data were recorded using a Peltier-cooled charge-coupled device (CCD) detector (1024 × 256 pixels). This system yielded a spectral resolution of about 1 cm^−1^. Silicon was used to calibrate the Raman setup in both Raman wavenumber and spectral intensity.

For pure myrrh, Raman spectra were recorded using an Echelle-type spectrometer Raman Flex 400 (Perkin Elmer, Rodgau, Germany) equipped with a thermoelectrically cooled CCD camera (cooled –50 °C) and a fiber-optic cable for excitation and collection of the Raman spectra. The 785 nm beam of the diode laser was used as the excitation source. The 180° scattering geometry was employed. The laser power at the sample was limited to 30 mW and the beam was focused to a spot with a diameter of 0.2 mm on the electrode. The integration time was 100 s.

### 2.12. Contact Angle on Melt Spun Multifilament Yarns

**Figure 1:**
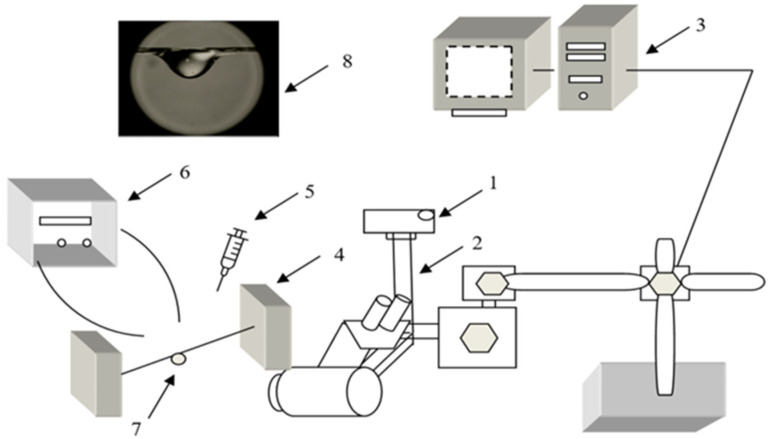
Principal scheme of equipment for measurement of yarn liquid contact angle: (1) Stereoscopic microscope, (2) digital camera, (3) computer, (4) yarn anchorage system, (5) pipette, (6) light source, (7) drop of liquid on the yarn, (8) picture of the video record [53].

In this study, the wettability of the yarns was characterized by measuring the contact angle using the static testing drop method. The absorbency tests using the drop method were performed with two types of liquids: physiological saline (B. Braun Melsungen AG, Hessen, Germany) and glycerol (Sigma–Aldrich, Chemie GmbH, Taufkirchen, Germany). This test method measures the liquid absorbency of yarn by determining the time required for a drop of liquid placed on the yarn surface to be completely absorbed into the yarn (Figure 1). A droplet of approximately ~9 µL, either water or glycerol (7), was deposited onto the sample yarn from a height of 0.2 cm using the needle (5). The time was recorded starting from when the droplet contacted the yarn and stopping when the reflection of light at the edge of the droplet disappeared. 

The liquid drop is completely absorbed by the yarn, or the variation is irrelevant. This is termed the drop absorbency time. The process was recorded using a stereo microscope (Nikon SMZ 800) and a Nikon Coolpix 4500 digital camera (1–2) connected to a computer (3). Changes in fluid angles in time were measured using the NIS element and compared with the initial wetting angles at the initial observation time of 0 s. The average liquid absorption time was calculated from 5 individual measurements.

### 2.13. In Vitro Degradation Test of PLA Melt Spun Multifilament Yarns

Degradation tests were performed only on PLA samples, as PP is nonbiodegradable. Sorensen buffer solution and degradation conditions were prepared, according to the standards ISO 13781:2017 [54]. For the accelerated degradation test, each sample was incubated at 55 °C. The temperature of 55 °C was chosen based on Van’t Hoff’s rule to create accelerated degradation conditions, enabling rapid degradation of the polymers. Each sample was immersed in 30 mL of Sørensen buffer at pH = 7.4. For each yarn type and time point, five specimens (*n* = 5) were degraded. The samples were shaken at 100 rpm at 55 °C, and the Sørensen buffer was replaced weekly. Samples were collected for analysis at the following time points: 0, 1, 2, 3, 4, 6, 8, 10, 12, 14, and 16 weeks. Each sample was washed three times with distilled water and dried at 40 °C before being weighed [55].

### 2.14. Antibacterial Properties of PLA Melt Spun Multifilament Yarns

The antimicrobial activity of the multifilament yarns samples was tested against Gram-negative bacteria *E. coli* (KMY1T) and Gram-positive bacteria *S. aureus* (ATCC25923). The bacteria were cultured at 37 °C in liquid Luria Broth (LB) (Carl Roth GmbH +Co. KG, Karlsruhe, Germany) medium (1.0 g tryptone, 0.5 g yeast extract powder, and 1 g NaCl dissolved in 100 mL of deionized water). All LB medium was autoclaved prior to the culturing. The control group used pure LB medium.

**Figure 2:**
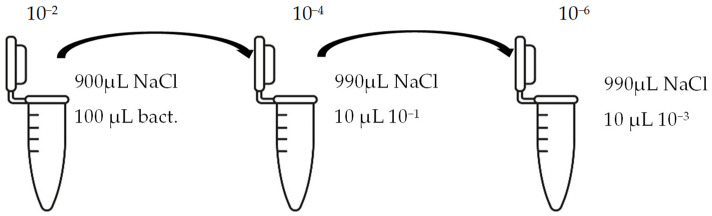
Bacterial suspension dilution scheme.

The suspension was incubated in an incubator (BIOSAN ES-20, Latvia) at 37 °C under mild shaking conditions (220 rpm) through the night. Bacteria culture was grown for 16–18 h. Bacterial growth was determined using the optical density (OD) value of the samples measured at 600 nm. After that, bacterial suspensions with an OD of 1 were prepared by diluting the bacterial culture in LB medium. These new bacterial suspensions were cultivated at 37 °C until the OD reached 0.6. Once the OD reached 0.6, multifilament yarns samples were added to the flasks and incubated until the OD of the suspensions reached 1. After that, the bacterial growth was stopped. The bacterial suspensions were diluted with autoclaved NaCl 0.9% solution, as presented in Figure 2, and then plated onto LB-agar plates.

Briefly, 50 µL of bacterial suspension at 10^−6^ dilution was distributed into LB-agar plates and incubated at 37 °C for 24 h in the thermostat. To evaluate the antimicrobial activities of the samples, the reduction in colony number between the group with multifilament yarns and control group after incubation was determined. The percentage reduction was calculated using the following equation:

$$C=\frac{A-B}{A}\times 100s$$

where C—the percentage reduction in bacterial colony numbers (%), and A and B — colony-forming units (CFU ml^−1^) recovered from the control group and group with multifilament yarns group, respectively, after inoculation and incubation. The bacterial colony was calculated using ImageJ (version 1.52av software).

## 3. Results

### 3.1. Estimation of Biological Active Compounds in Ethanolic Myrrh Extract

**Figure 3:**
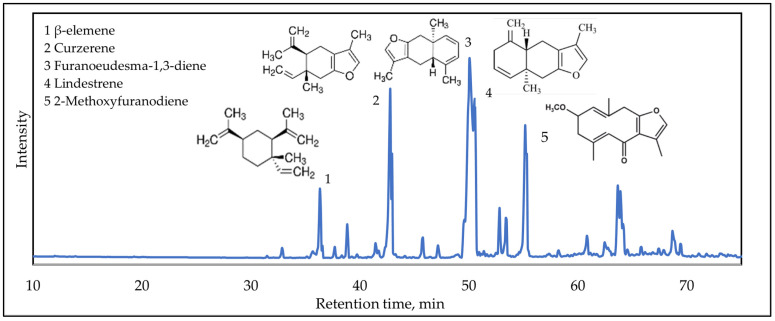
GC/MS chromatograms of ethanolic myrrh extract.

Myrrh resin is rich in organic and inorganic elements and volatile oils, which help in healing skin ulcers and sores [45]. Gas chromatography–mass spectrometry (GC–MS) analysis was used to investigate the compounds of ethanolic myrrh resin extract, and the results are presented in Figure 3. The major peaks of the chromatogram in Figure 3 are characteristic for the major compounds found in myrrh: (1) β-elemene, (2) curzerene, (3) lindestrene, (4) furanoeudesma-1,3-diene, and (5) 2-methoxyfuranodiene. Curzerene is a type of terpenoid; sesquiterpenes, like furanoeudesma-1,3-diene and lindestrene, a possible antiviral compound, have antibacterial, anti-inflammatory, and antiviral effects and analgesic properties [56,57]. The results correlated with other research studies where major terpenoids were identified [58,59].

### 3.2. The Influence of Myrrh Extract on the Linear Density and Mechanical Properties of Melt Spun Multifilament Yarns

**Figure 4:**
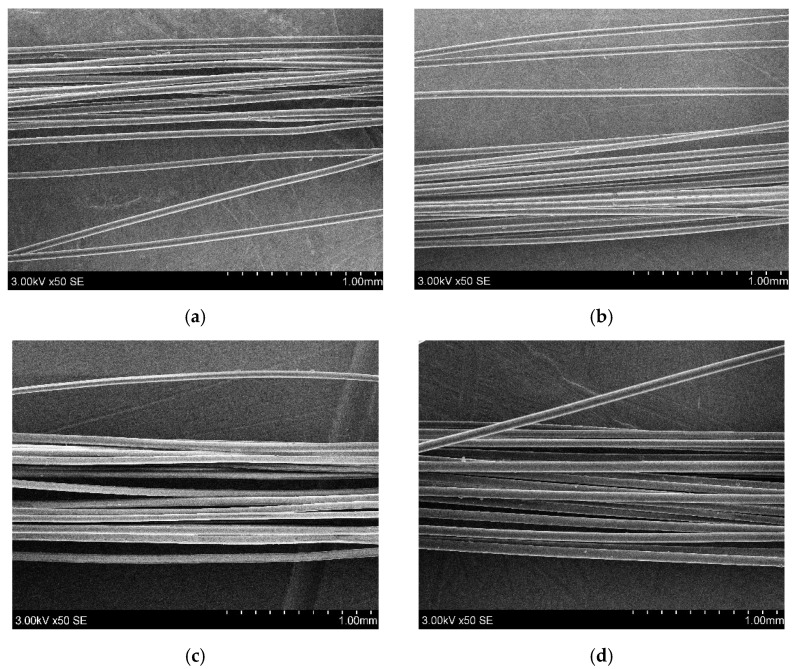
SEM images of (**a**) PLA3, (**b**) PLA/M3, (**c**) PP3, and (**d**) PP/M3.

SEM images of pure PLA3, PP3 and PLA/M3, PP/M3 melt-spun multifilament yarns are presented in Figure 4. Melt spun yarns have a smooth, homogenous surface without cracks or visible myrrh derivatives (Figure 4b: PLA/M3 and Figure 4d: PP/M3). These results correlate with other works. Yu and coauthors [60] formed composite PLA filaments with 3% and 6% talc additive. The obtained composite filaments were homogenous and had a smooth surface.

**Figure 5:**
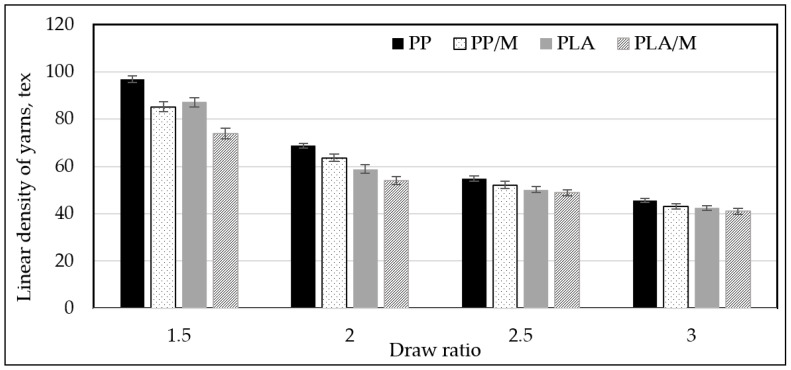
The dependence on linear density of PP, PP/M, PLA, and PLA/M melt spun yarns at different draw ratios (DRs).

In Figure 5, the variation in the linear density (tex) of melt spun PP and PLA yarns is presented. It can be stated that thinner filaments are formed from PP/M and PLA/M than from pure PP and PLA. The linear density (tex) of PP/M filaments is smaller, by 12%, 7%, 5%, and 5%, than pure PP, while PLA/M is smaller, by 20%, 7%, 3%, and 4%, than pure PLA, at drawing ratios (DRs) of 1.5; 2; 2.5, and 3, respectively. Myrrh extract had a significant influence on the linear density of multifilament yarns when they were formed at the lowest DR—1.5. An increase in DR (from 1.5 to 3) resulted in a decrease in the linear density of all yarns to about 50%. The DR influences the properties of the yarn due to changes in polymer alignments and crystallinity, as well as the linear density of yarns. An increase in DR resulted in an increased alignment of polymer chains in the drawing direction, while a low DR resulted in a very brittle yarn with a high linear density. The results correlate with other research studies [61,62,63].

**Figure 6:**
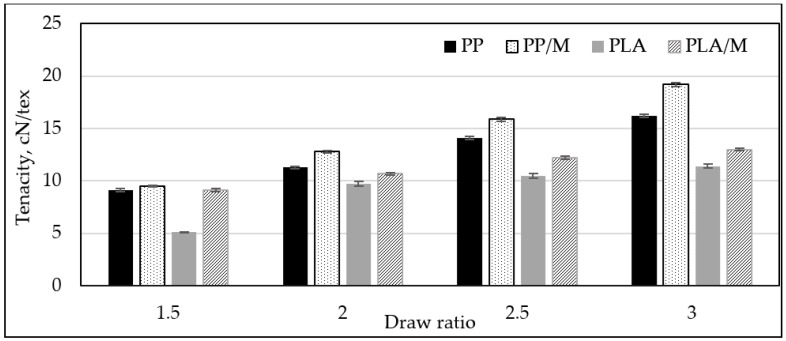
The dependence of the tenacity of melt spun yarns PP, PP/M, PLA, and PLA/M on different draw ratio (DR).

**Figure 7:**
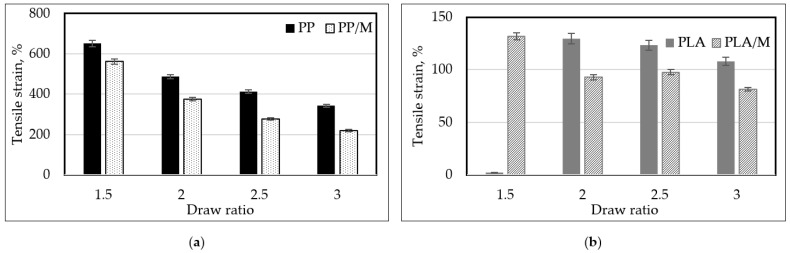
The dependence of the tensile strain of melt spun yarns ((**a**)—PP, PP/M; (**b**)—PLA, PLA/M) on different draw ratios (DRs).

The tenacity (cN/tex) of the formed melt spun yarn is presented in Figure 6. The melt spun yarns formed at higher DR were characterized by higher tenacity (cN/tex). Increasing the DR from 1.5 to 3 resulted in an increase in the tenacity of all melt spun yarns. The tenacity (cN/tex) of PP3, PP/M3, PLA3, and PLA/M3 increased by 43%, 50%, 52%, and 25%, respectively. The tensile strains of the different melt spun yarns are presented in Figure 7. It can be observed that an increase in DR leads to a decrease in the tensile strain (%) for all yarn types. The tensile strain (%) of melt spun PP3, PP/M3, and PLA/M3 yarns decreased by 47%, 60%, and 38%, respectively, when the DR was increased from 1.5 to 3. S. Noh and coauthors [12] analyzed the effect of draw ratio on the mechanical properties of PLA melt spun yarns and stated that specific strength (cN/tex) increased as elongation at break (%) decreased with an increasing draw ratio.

Melt spun yarns containing myrrh had higher tenacity (4–15%) and lower tensile strain (13–35%) than pure PP or PLA yarns. It should be noted that at a DR of 1.5, PLA/M showed 43% higher tenacity (cN/tex) compared to pure PLA. This indicates that at this DR, pure PLA yarns were very brittle, complicating the analysis of their mechanical properties.

According to the presented results, it is possible to make an assumption that at a higher draw ratio, the presence of myrrh increases chain mobility and induces crystallization (Table 2); respectively, yarns with myrrh and at higher draw ratio have higher tenacity (cN/tex) and lower tensile strain (%).

### 3.3. Optical Analysis of Melt Spun Multifilament Yarns

**Figure 8:**
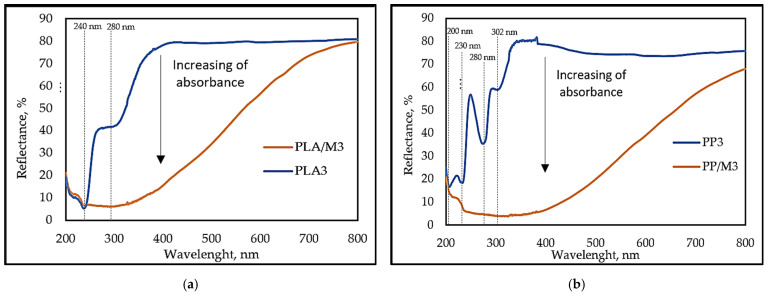
UV–Vis spectra of (**a**) PLA3 and PLA/M3; (**b**) PP3 and PP/M3.

UV–Vis spectroscopy analysis was used to investigate the changes in light reflectance of pure yarns and myrrh resin multifilament yarns. It is shown in Figure 8a that for pure PLA3, light reflectance decreases with decreasing wavelength. The reflectance begins to decrease at 370 nm and reaches a minimum at 240 nm. The first minimum is observed at approximately 300 nm. This peak could be attributed to ethylene copolymer additive in PLA, which improves the toughness, flexibility, and impact strength of PLA parts produced by the thermoforming and injection molding techniques [64]. The second peak of pure PLA3 reflectance is at 240 nm and is due to the strong absorption of UV radiation by the ester groups in the PLA polymer chain [65].

Meanwhile, from the UV–Vis spectra of pure PP3 (Figure 8b), it can be observed that the reflectance started decreasing at 360 nm. The reflectance minima (the peak of absorbance) at around 200 nm and 230 nm are typical for π→π* electronic transitions in CC double bonds in polymer chains [66,67]. The absorbance at 280 nm is, rather, due to the nπ* transition in carbonyl compounds [66]. A peak at 300 nm could be a UV-stabilizer, which usually has a peak at 290–300 nm [68].

PLA/M3 and PP/M3 (DR-3) yarns showed a significant increase in absorbance (decrease in diffuse reflectance) in the wavelength range of 750 to 400 nm, along with broad and intensive absorption bands spanning the entire UV region (from 200 to 400 nm). PP/M3 yarns showed approximately 10% lower diffuse reflectance across the entire visible light region compared to PLA/M3 yarns. The yarns with myrrh (PP/M3 and PLA/M3) showcased higher light absorption than pure (PP3 and PLA 3) yarns, likely caused by the myrrh coating. Myrrh resin has various volatile oils (elemol, eugenol, limonene, esters, etc.) and alcohol-soluble resins (commiphorinic acids, commiphoric acids, commiferin, heerabomyrrhols, etc.). Most of them are strong light-absorbers [45,69].

Multifilament yarns PLA/M3 and PP/M3 are characterized by a lower reflectance in the visible and ultraviolet regions, which was caused by myrrh volatile oils due to scattered light. The obtained results show that the addition of myrrh, which contains organic compounds, led to an increase in transmittance. These findings correlate with the results of other research that investigated the effects of adding organic compounds like curcumin, cinnamon oil, or wine seed extract to the polymer matrices [37,70,71].

### 3.4. Thermal Behavior of Melt Spun Multifilament Yarns

**Figure 9:**
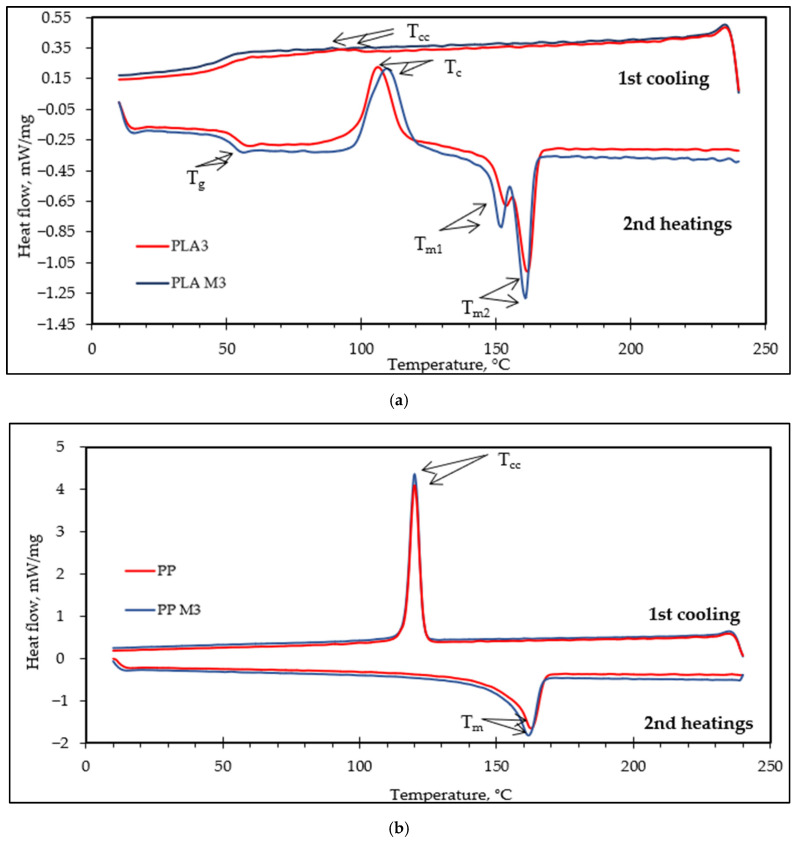
DSC thermograms of (**a**) PLA3 and PLA/M3; (**b**) PP3 and PP/M3.

The thermal characteristics and crystallization behavior of PLA3, PLA/M3, PP3, and PP/M3 multifilament yarns were investigated using DSC. A higher draw ratio facilitates orientation-induced crystallization of the PLA chains, thereby improving the thermal properties of the PLA fibers [12]. For this reason, the influence of myrrh on thermal properties of melt spun yarns was analyzed at the highest drawing ratio (DR: 3). The corresponding thermograms are presented in Figure 9. The values of glass transition temperature (T_g_), crystallization temperature (T_c_), exothermic enthalpy of crystallization ∆Hc (J/g), melting temperatures T_m1_ and T_m2_, cold crystallization temperature (T_cc_), and degree of crystallization (X_c_) of the multifilament yarns are given in Table 2.

**Table 2:** DSC analysis of pure PLA and PLA with myrrh resin multifilament yarns.

Code of Sample	T_g_ (°C)	T_c_ (°C)	∆H_c_ (J/g)	T_m1_ (°C)	T_m2_ (°C)	T_cc_ (°C)	∆H _m_ (J/g)	Crystallinity X_c_ (wt%)
PLA3	57.1	106	34.9	153.7	161.5	49.4	44.7	11
PLA/M3	56.3	109.4	43.1	151.6	160.7	48.1	54.0	13
PP3	-	-	-	-	162.9	120.1	102.9	49
PP/M3	-	-	-	-	161.9	120.1	102.5	55

When analyzing the data for PLA multifilament yarns from Table 2, it is evident that myrrh resin has no significant influence on T_g_ (with a difference of 0.6 °C) or T_m_ (with a difference of 1–2 °C). One of the most important features of polymers is the degree of polymer crystallinity. This refers to the overall level of crystalline components relative to the amorphous component. The percent of crystallinity is related to many of the properties of the PLA polymer, such as brittleness, toughness, modulus, optical clarity, and creep [72]. By analyzing the thermograms of the melt spun multifilament yarns, it is observed that the multifilament yarns from PLA/M3 have a higher degree of crystallinity (X_c_) than those of PLA3. The exothermal crystallization temperature (T_c_) for PLA3 was slightly lower (106 °C) compared to PLA/M3 (109.4 °C). Biswal and coauthors [12] analyzed the effect of essential oils on the thermal properties of PLA microparticles and stated that thymol and other essential oils decreased crystallization temperature after the second round of heating [73].

When analyzing the data for PP3 multifilament yarns from Table 2, it is evident that there was no significant difference between T_m2_, T_cc_, and H_m_ of PP3 and PP/M3 yarns. Myrrh had a significant influence on the increase in crystallinity degree of PP/M3 yarns (from 49% for PP3 to 55% for PP/M3). This indicates that myrrh is a good nucleating agent for PP polymer. Carrier talc did not influence T_m_ or T_c_ but resulted in an increase in X_c_ of PP films in the study [74]. However, contrary to our results, carrier talc had a significant influence on the decrease in enthalpy.

Figure 9b presents the thermograms of pure PP3 and PP/M3. It can be concluded that the PP3 samples, as well as the PP3 samples after the addition of myrrh, demonstrated similar thermal behaviors. There was a notable reduction in melting point (T_m_).

### 3.5. Raman Spectroscopy of Melt Spun Multifilament Yarns

**Figure 10:**
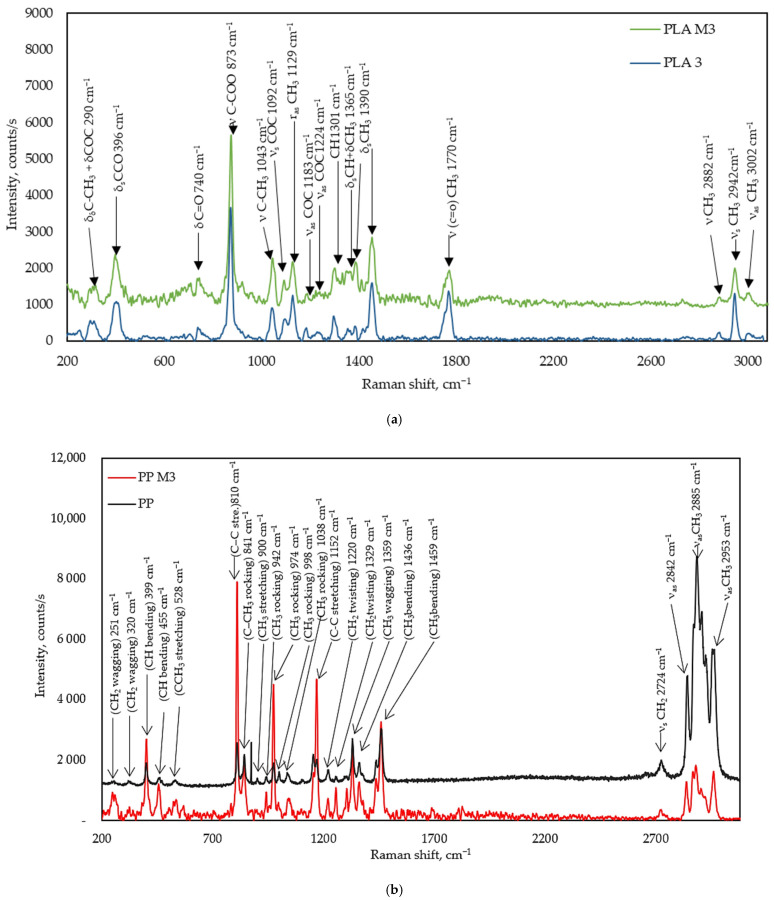
Raman spectra of (**a**) pure PLA3 and PLA/M3; (**b**) pure PP3 and PP/M3.

Figure 10a shows the Raman spectra of pure PLA3 multifilament yarns and PLA/M3, highlighting the main bands and their observed wavenumbers. A comparison of pure PLA3 and PLA/M3 revealed the typical peaks of PLA. Peaks at 3002, 2949, and 2882 cm^−1^ were assigned to the CH_3_ group stretching vibration; asymmetric stretching 2995–3002 cm^−1^; symmetric stretching 2944–2947 cm^−1^ and 2878–2889 cm^−1^ modes [75]. The second peak of pure PLA corresponds to the C=O group stretching at 1770, 1454, and 1390 cm^−1^ symmetric deformation modes of the CH_3_ groups. The peak at 1365 cm^−1^ could be due to the symmetric deformation modes of the CH_3_ group. The peak at 1224 cm^−1^ is an asymmetric vibration band of C-O-C. The peak at 1301 cm^−1^ is likely due to the CH symmetric deformation peak that is usually found between 1299 and 1305 cm^−1^. The peak at 1129 cm^−1^ could be assigned to the CH_3_ group rocking vibration and the peak at 1043 cm^−1^ to the stretching of the C-CH_3_ bond, while the peaks at 873 cm^−1^ and 396 cm^−1^ could be assigned to the vibrational states of the C-COO and C-CO groups, respectively. The 740 cm^−1^ peak showed the rocking vibration of C=O. The peak at 290 cm^−1^ is characteristic of the vibration of two different groups: C-O-C and C-CH_3_ [76,77,78].

A comparison of pure PP3 and PP with myrrh resin (PP/M3) revealed the typical peaks of PP. The peak at 2953 cm^−1^ is asymmetric CH_3_ stretching vibration, while peaks at 2885 and 2842 cm^−1^ are for symmetric CH_3_ stretching. The peak at 2774 cm ^−1^ is a symmetric CH_2_ stretching vibration. Raman peaks at the 1436 and 1459 cm^−1^ regions can be assigned to the asymmetric bending vibration mode of the -CH_3_ group. The band displayed at 1359 cm^−1^ is related to the -CH_3_ symmetric bending vibration mode. The bending mode of the CH and the twisting mode of the -CH_2_- group are assigned to 1329 cm^−1^. The bands at 1220 cm^−1^ are allocated to the stretching of the C-C backbone. The band displayed at 1359 cm^−1^ is related to the -CH_3_ symmetric bending vibration mode at 1152 cm^−1^ (C-C stretching) and CH bending at 1038 cm^−1^ (CH bending). The peaks at 998, 974, and 942 cm^−1^ are for rocking stretching. At 900 cm^−1^ is CH_3_ stretching, 841 cm^−1^ is C-CH_3_ rocking, 810 cm^−1^ is C-C stretching, and CCH_3_ stretching is at 528 cm^−1^. CH_2_ wagging and CH bending are at 320 and 455 cm^−1^, and CH_2_ wagging and CH bending are at 251 and 399 cm^−1^ [79,80,81].

**Figure 11:**
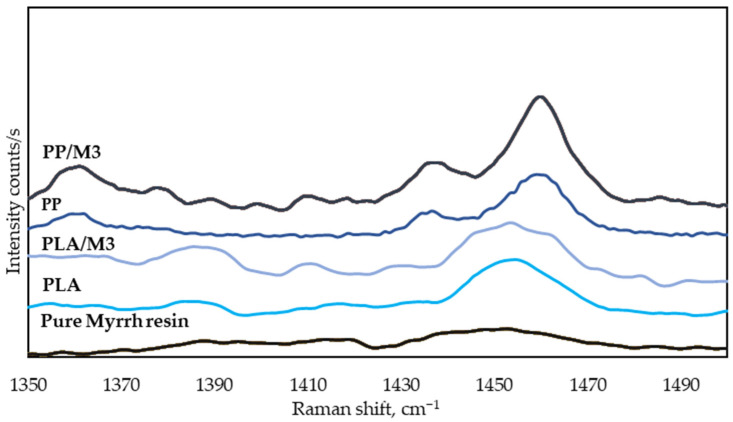
Comparative Raman spectra of the wavenumber region 1350–1500 cm^−1^.

As mentioned earlier, myrrh contains a lot of terpenoid sesquiterpenes, such as curzerene, lindestrene, and furanoeudesma-1,3-diene. These compounds generated peaks in the 1420–1460 cm^−1^ range, corresponding to the β(CH)_2_ scissoring vibration [82,83]. Moreover, in this region, there are very strong signals for the δCH_3_ asymmetric vibration at 1458 cm^−1^ and a weaker δCH_3_ asymmetric vibration at 1435 cm^−1^ [68]. PLA also shows a strong asymmetric δCH_3_ vibration at 1454 cm^−1^, as seen in Figure 11. The signal for pure myrrh resin is weaker than those for PP or PLA. We can assume that the stronger signals for PP or PLA overshadow the sesquiterpenes signal.

### 3.6. Contact Angle on Melt Spun Multifilament Yarns

The contact angles on melt spun yarns of the investigated liquids (physiological saline and glycerol) are listed in Table 3. As can be seen in the table, different liquids have different contact angles due to their different surface energies, which are influenced by their chemical groups, with glycerol having a slightly higher surface tension (i.e., 76.2 mN/m) than physiological saline (i.e., 73.9 mN/m). Glycerol has a higher contact angle, regardless of the drawing ratio or material, when compared with physiological saline. Yarns with higher linear density, formed at a lower drawing ratio (PLA1.5, PLA1.5M, PP1.5, and PP1.5M), exhibit a higher surface area and smaller contact angles for the analyzed liquids. The contact angles of melt spun yarns at a DR of 1.5 are 10–25% smaller than those of yarns formed at a DR of 3 when using physical saline. With glycerol, the contact angles on the same melt spun yarns are 4–10% smaller. 

The presence of myrrh in multifilament yarns reduces their wettability, as the contact angles of physiological saline and glycerol are 6–18% higher. These results correlate with findings from studies analyzing PLA films with curcumin [68] or limonene [84]. The addition of hydrophobic materials to another hydrophobic material, such as PLA, results in a composite with a higher liquid contact angle.

### 3.7. In Vitro Degradation Test of PLA Melt Spun Multifilament Yarns

**Figure 12:**
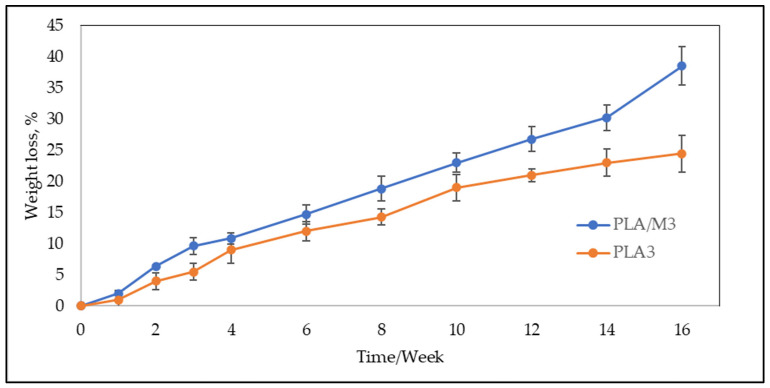
Weight loss of PLA3 and PLA/M3 multifilament yarns.

To evaluate the effect of myrrh on the degradation of PLA multifilament yarns, the mass loss of PLA3 and PLA/M3 melt spun multifilament yarns was analyzed over a 3-month period. It is known that the diameter of yarns has a significant influence on the degradation time, with thicker yarns generally exhibiting longer degradation time [85]. In this study, PLA3 and PLA/M3 yarns were analyzed, with a linear density of 42.5 ± 0.9 tex and 41 ± 1.5 tex, and a diameter of 44.4 ± 2.4 µm and 43.4 ± 2.2 µm, respectively. From the results presented in Figure 12, it can be seen that PLA/M3 yarns had a higher weight loss over the observation period. During the first four weeks, both types of multifilament yarns decreased in weight by 10%. Beyond this initial period, the difference in weight loss between the analyzed yarns continued to increase. The PLA3 multifilament yarns lost 20% of their initial weight after 12 weeks, while PLA/3M lost the same amount after 8 weeks. After 16 weeks, PLA3 yarns had lost 25% of their weight, while PLA/3M had lost 38%. This suggests that myrrh resin is present within the PLA matrix, as the degradation process began earlier, likely due to the acidity effect of myrrh [86]. All these results confirm that myrrh increases the degradation of PLA melt spun yarns.

### 3.8. Antibacterial Properties of PLA Melt Spun Multifilament Yarns

Myrrh contains various compounds, with about 300 molecules already described in plants of the *Commiphora* genus. The predominant classes of these compounds are sesquiterpenes (eudesmanes, elemanes, germacranes, and cadinanes, including furanoeudesma-1,3-diene, curzerene, and lindestrene) and triterpene [56]. GC–MS analysis identified the same compounds (β-elemene, curzerene, lindestrene, furanoeudesma-1,3-diene, and 2-methoxyfuranodiene) in the prepared ethanolic myrrh extract (see Section 2.1).

The antimicrobial activities of melt spun yarns were evaluated against pathogenic bacterial strains, including both Gram-positive (*S. aureus*) and Gram-negative (*E. coli*) bacteria. Melt spun yarns with myrrh did not show any antibacterial activity against *E. coli*. This may be due to the differences in the cell wall structures of Gram-negative bacteria, which vary chemically and structurally, depending on the strain (e.g., outer and inner membranes, peptidoglycan layer, lipopolysaccharides, etc.) [87].

A different situation was seen when analyzing Gram-positive bacteria. As shown in Table 4, PLA/M3 yarns inhibit the proliferation of *S. aureus*, achieving an approximately 84.42% reduction in bacteria colony counts compared to pure PLA multifilament yarns.

Although the antibacterial effects of essential oils are often attributed to their main components, studies have shown that this is not always the case. For example, curzerene, a terpenoid with antioxidant properties, is known to be effective against diseases related to oxidative damage. However, terpenes are not generally considered to be a group of compounds with strong natural antimicrobial activity [58,87].

## 4. Conclusions

This study investigated the effect of myrrh resin on the properties of PP and PLA melt spun multifilament yarns. It was determined that the presence of myrrh resin led to significant changes in the mechanical, physical, and antibacterial properties of the yarns. Multifilament yarns with myrrh resin exhibited slightly higher degrees of crystallinity (%), which contributed to their higher tenacity (cN/tex) and lower tensile strain (%) compared to pure PLA and PP yarns. PLA/M and PP/M multifilament yarns demonstrated the highest absorbance across the entire UV region compared to pure PLA and PP yarns, likely due to organic compounds in myrrh resin, which increase transmittance. The presence of myrrh in multifilament yarns reduces wettability but increases the degradation rate of biodegradable multifilament yarns. Melt spun yarns with myrrh did not show any antibacterial activity against Gram-negative bacteria (*E. coli*) but reduced the proliferation of Gram-positive bacteria (*S. aureus*).

This study provides insights into the development of antibacterial melt spun yarns using natural myrrh resin. The observed changes in the structural, mechanical, physical, and antibacterial properties show the potential of myrrh resin for use in yarns for various applications in healthcare textile products. Further research must be carried out for broader analysis of antibacterial properties and increasing the wetting behavior of meltspun yarns with natural myrrh derivatives.

## Figures and Tables

**Table 1:** Parameters for spinning to obtain multifilament yarns.

Code	Samples	Temperature at Heating Zones, °C	Speed of the Stretching Rolls, rpm				Drawing Ratio
			S1	S2	S3	S4	
PP1.5	PP	205	100	116	139	150	1.5
PP/M1.5	PP + 10 wt% myrrh resin						
PP2	PP		100	134	169	201	2
PP/M2	PP + 10 wt% myrrh resin						
PP2.5	PP	205	100	150	204	251	2.5
PP/M2.5	PP + 10 wt% myrrh resin						
PP3	PP		100	168	237	301	3
PP/M3	PP + 10 wt% myrrh resin						
PLA1.5	PLA	205	100	116	139	150	1.5
PLA/M1.5	PLA+ 10 wt% myrrh resin						
PLA2	PLA		100	134	169	201	2
PLA/M2	PLA+ 10 wt% myrrh resin						
PLA2.5	PLA	205	100	150	204	251	2.5
PLA/M2.5	PLA+ 10 wt% myrrh resin						
PLA3	PLA		100	168	237	301	3
PLA/M3	PLA+ 10 wt% myrrh resin						

**Table 3:** The values of contact angle of liquids (physical saline and glycerol) on melt spun multifilament yarns.

Liquid	Phys. Saline	Glycerol	Phys. Saline	Glycerol	Phys. Saline	Glycerol	Phys. Saline	Glycerol	Phys. Saline	Glycerol
Time	0		10		20		60		120	
Samples										
PLA1.5	52.2 ± 1.4	81.1 ± 3.4	-	75.5 ± 3.6	-	73.9 ± 4.7	-	73.4 ± 4.0	-	72.2 ± 5.0
PLA/M1.5	62.7 ± 2.7	85.4 ± 5.9	50.9 ± 4.7	83.1 ± 8.5	40.5 ± 5.6	81.9 ± 8.9	-	80.9 ± 8.2	-	80.1 ± 7.2
PLA3	71.4 ± 1.6	87.6 ± 7.9	63.9 ± 4.0	83.1 ± 8.5	55.1 ± 4.1	81.9 ± 8.9	42.2 ± 6.5	80.9 ± 8	-	80.1 ± 7.2
PLA/M3	69.6 ± 8.3	88.2 ± 5.5	63.6 ± 8.1	84.3 ± 6.1	54.7 ± 7.4	83.9 ± 5.3	33.1 ± 8.7	82.9 ± 6.1	-	81.9 ± 6.9
PP1.5	54.8 ± 3.3	78.4 ± 5.2	34.8 ± 2.7	77.9 ± 5.3	18.1 ± 0.3	74.9 ± 3.2	-	71.9 ± 3	-	71.4 ± 2.4
PP/M1.5	62.5 ± 1.9	79.6 ± 0.9	34.2 ± 4.6	76.1 ± 2	22.9 ± 2.1	75.4 ± 2.4	-	72.2 ± 2.7	-	72.1 ± 2.8
PP3	60.6 ± 2.7	85.4 ± 5.7	50.3 ± 4.1	81.7 ± 7.5	42.9 ± 3.4	78.9 ± 6.7	37.3 ± 2.3	77.1 ± 5.3	-	77.0 ± 6.1
PPM3	64.7 ± 5.1	92.5 ± 2.9	54.9 ± 6.7	88.5 ± 7.4	43.6 ± 1.1	88.1 ± 5.7	30.7 ± 5.5	87.9 ± 5.1	-	86.7 ± 4.8

**Table 4:** Antibacterial activity of PLA/M3 and PLA3 melt spun multifilament yarns.

*S. aureus*, Bacteria Reduction = 84.42%		*S. aureus* Bacteria Reduction Didn’t Indicated	
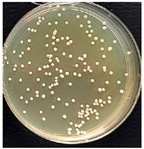	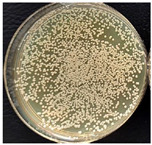	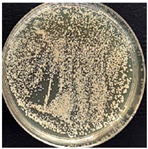	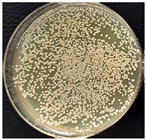
PLA/M3, 10^−6^	Control, 10^−6^	PLA3, 10^−6^	Control, 10^−6^

## Data Availability

The original contributions presented in the study are included in the article, further inquiries can be directed to the corresponding author.

## References

[1] Tabor J., Thompson B., Agcayazi T., Bozkurt A., Ghosh T.K. (2022). Melt-Extruded Sensory Fibers for Electronic Textiles. Macromol. Mater. Eng..

[2] Santos D.M., Correa D.S., Medeiros E.S., Oliveira J.E., Mattoso L.H.C. (2020). Advances in Functional Polymer Nano fibers: From Spinning Fabrication Techniques to Recent Biomedical Applications. ACS Appl. Mater. Interfaces.

[3] Kopf S., Åkesson D., Skrifvars M. (2022). Textile Fiber Production of Biopolymers—A Review of Spinning Techniques for Polyhydroxyalkanoates in Biomedical Applications. Polym. Rev..

[4] Oh T.H. (2006). Melt spinning and drawing process of PET side-by-side bicomponent fibers. J. Appl. Polym. Sci..

[5] Zhang Z., Zhou J., Yu S., Wei L., Hu Z., Xiang H., Zhu M. (2023). Melt-spun bio-based PLA-co-PET copolyester fibers with tunable properties: Synergistic effects of chemical structure and drawing process. Int. J. Biol. Macromol..

[6] Xie K., Xu S., Xu K., Zhang W., Yu S., Wang P., Han Z., He N., Chen P. (2023). Preparation and antibacterial properties of copper phthalate/polyethylene terephthalate composition fiber. Alex. Eng. J..

[7] Seyhan A., Gunaydin B.N., Polat Y., Kilic A., Demir A., Avci H. (2022). Improvement of polyethylene fiber wettability and mechanical properties through an environmentally sustainable spinning process. Int. J. Adhes. Adhes..

[8] Kanerva M., Puolakka A., Takala T.M., Elert A.M., Mylläri V., Jönkkäri I., Sarlin E., Seitsonen J., Ruokolainen J., Saris P. (2019). Antibacterial polymer fibres by rosin compounding and melt-spinning. Mater. Today Commun..

[9] Liang J.Z. (2020). Melt strength and stretching ratio of low-density polyethylene composites loaded with nanoscale zinc oxide. Adv. Ind. Eng. Polym. Res..

[10] Gaidukovs S., Lyashenko I., Rombovska J., Gaidukova G. (2016). Application of amber filler for production of novel polyamide composite fiber. Text. Res. J..

[11] Demirhan A.D., Tuğral S., Sarıışık M., Göktaş D., Kartal G.E. (2024). Investigation of polypropylene yarns containing silver nanoparticles: Fiber characteristics, yarn properties, and fabric performance analysis. J. Appl. Polym. Sci..

[12] Noh S., Jung W., Sim S., Son H.S., Choi J.H., Koo J. (2023). Effect of Drawing Conditions on Crystal Structure and Mechanical Properties of Melt-Spun Polylactic Acid Fibers. Fibers Polym..

[13] Xue W., Chen P., Wang F., Wang L. (2019). Melt spinning of nano-hydroxyapatite and polycaprolactone composite fibers for bone scaffold application. J. Mater. Sci..

[14] Shi X.Q., Ito H., Kikutani T. (2005). Characterization on mixed-crystal structure and properties of poly(butylene adipate-co-terephthalate) biodegradable fibers. Polymer.

[15] Saigusa K., Takarada W., Kikutani T. (2020). Improvement of the Mechanical Properties of Poly(Glycolic Acid) Fibers Through Control of Molecular Entanglements in the Melt Spinning Process. J. Macromol. Sci. Part B Phys..

[16] Gahleitner M., Paulik C. (2012). Polypropylene and Other Polyolefins.

[17] Tsioptsias C., Leontiadis K., Tzimpilis E., Tsivintzelis I. (2021). Polypropylene nanocomposite fibers: A review of current trends and new developments. J. Plast. Film Sheeting.

[18] Adomavičiūtė E., Baltušnikaitė-Guzaitienė J., Juškaitė V., Žilius M., Briedis V., Stanys S. (2018). Formation and characterization of melt-spun polypropylene fibers with propolis for medical applications. J. Text. Inst..

[19] Radheshkumar C., Münstedt H. (2006). Antimicrobial polymers from polypropylene/silver composites-Ag+ release measured by anode stripping voltammetry. React. Funct. Polym..

[20] Erem A.D., Ozcan G., Skrifvars M. (2013). In vitro assessment of antimicrobial polypropylene/zinc oxide nanocomposite fibers. Text. Res. J..

[21] Esthappan S.K., Kuttappan S.K., Joseph R. (2012). Thermal and mechanical properties of polypropylene/titanium dioxide nanocomposite fibers. Mater. Des..

[22] Dastjerdi R., Mojtahedi M.R.M. (2013). Multifunctional melt-mixed Ag/TiO_2_ nanocomposite PP fabrics: Water vapour permeability, UV resistance, UV protection and wear properties. Fibers Polym..

[23] Dabrowska I., Fambri L., Pegoretti A., Slouf M., Vackova T., Kolarik J. (2015). Spinning, drawing and physical properties of polypropylene nanocomposite fibers with fumed nanosilica. Express Polym. Lett..

[24] Kara S., Ureyen M.E., Erdogan U.H. (2016). Structural and Antibacterial Properties of PP/CuO Composite Filaments Having Different Cross Sectional Shapes. Int. Polym. Process..

[25] Broda J., Slusarczyk C., Fabia J., Demsar A. (2016). Formation and properties of polypropylene/stearic acid composite fibers. Text. Res. J..

[26] Fredi G. (2019). Melt-spun polypropylene filaments containing paraffin microcapsules for multifunctional hybrid yarns and smart thermoregulating thermoplastic composites. Express Polym. Lett..

[27] Cayla A., Campagne C., Rochery M., Devaux E. (2012). Melt spun multifilament yarns of carbon nanotubes-based polymeric blends: Electrical, mechanical and thermal properties. Synth. Met..

[28] Qi Z., Wang B., Sun C., Yang M., Chen X., Zheng D., Yao W., Chen Y., Cheng R., Zhang Y. (2022). Comparison of Properties of Poly(Lactic Acid) Composites Prepared from Different Components of Corn Straw Fiber. Int. J. Mol. Sci..

[29] Devaux E., Aubry C., Campagne C., Rochery M. (2011). PLA/Carbon Nanotubes Multifilament Yarns for Relative Humidity Textile Sensor. J. Eng. Fibers Fabr..

[30] John M.J., Anandjiwala R., Oksman K., Mathew A.P. (2012). Melt-Spun Polylactic Acid Fibers: Effect of Cellulose Nanowhiskers on Processing and Properties. J. Appl. Polym. Sci..

[31] Kanerva M., Mensah-Attipoe J., Puolakka A., Takala T.M., Hyttinen M., Layek R., Palola S., Yudin V., Pasanen P., Saris P. (2021). Weathering of antibacterial melt-spun polyfilaments modified by pine rosin. Molecules.

[32] Sonseca A., Madani S., Muñoz-Bonilla A., Fernández-García M., Peponi L., Leonés A., Rodríguez G., Echeverría C., López D. (2020). Biodegradable and Antimicrobial PLA–OLA Blends Containing Chitosan-Mediated Silver Nanoparticles with Shape Memory Properties for Potential Medical Applications. Nanomaterials.

[33] Malafeev K.V., Moskalyuk O.A., Yudin V.E., Elena N., Ivan E.M., Gordina E.M., Bozhkova S.A., Ivan E.M., Gordina E.M., Bozhkova S.A. (2022). Effects of silver nanoparticle on mechanical properties of polylactide composite yarns with different structure. J. Text. Inst..

[34] Palanikumar L., Ramasamy S.N., Balachandran C. (2014). Size-dependent antimicrobial response of zinc oxide nanoparticles. IET Nanobiotechnol..

[35] Mohsenpour H., Pesce M., Patruno A., Bahrami A., Pour P.M., Farzaei M.H. (2021). A review of plant extracts and plant-derived natural compounds in the prevention/treatment of neonatal hypoxic-ischemic brain injury. Int. J. Mol. Sci..

[36] Chirila L., Constantinescu G.C., Danila A., Popescu A., Constantinescu R.R., Săndulache I.M. (2020). Functionalization of textile materials with bioactive polymeric systems based on propolis and cinnamon essential oil. Ind. Textila.

[37] Moraczewski K., Pawłowska A., Stepczyńska M., Malinowski R., Kaczor D., Budner B., Gocman K., Rytlewski P. (2020). Plant extracts as natural additives for environmentally friendly polylactide films. Food Packag. Shelf Life.

[38] Salmas C.E., Giannakas A.E., Baikousi M., Leontiou A., Siasou Z., Karakassides M.A. (2021). Development of Poly(L-Lactic Acid)/Chitosan/Basil Oil Active Packaging Films via a Melt-Extrusion Process Using Novel Chitosan/Basil Oil Blends. Processes.

[39] Llana-Ruiz-Cabello M., Pichardo S., Bermúdez J.M., Baños A., Núñez C., Guillamón E., Aucejo S., Cameán A.M. (2016). Development of PLA films containing oregano essential oil (*Origanum vulgare* L. virens) intended for use in food packaging. Food Addit. Contam.-Part A Chem. Anal. Control Expo. Risk Assess..

[40] Ahmed J., Mulla M.Z., Arfat Y.A. (2016). Thermo-mechanical, structural characterization and antibacterial performance of solvent casted polylactide/cinnamon oil composite films. Food Control.

[41] Bolskis E. (2022). Formation and Investigation of Mechanical, Thermal, Optical and Wetting Properties of Melt-Spun Multifilament Poly (lactic acid) Yarns with Added Rosins. Polymers.

[42] Bolskis E., Adomavičiūtė E., Griškonis E., Norvydas V. (2020). Influence of Myrrh Extracts on the Properties of PLA Films and Melt-Spun Multifilament Yarns. Materials.

[43] Massoud A., El Sisi S., Salama O., Massoud A. (2001). Preliminary study of therapeutic efficacy of a new fasciolicidal drug derived from Commiphora molmol (MYRRh). Am. J. Trop. Med. Hyg..

[44] Mansouri R.A., Ahmad A., Roushdy M.M., Alshaibi H.F., Ragab M. (2023). Pharmacological Studies on the Antidiabetic, Antioxidant, and Antimicrobial Efficacies of Commiphora myrrha Resin in Streptozotocin-Induced Diabetes in Rats: A Preclinical Study. J. Diabetes Res..

[45] Shameem I., Fahad T. (2018). Phytochemical & therapeutic potentials of Murr makki (*Commiphora myrrha*): A review. Indian J. Appl. Res..

[46] Ajiteru O., Lee O.J., Kim J.H., Lee Y.J., Lee J.S., Lee H., Sultan M.T., Park C.H. (2022). Fabrication and characterization of a myrrh hydrocolloid dressing for dermal wound healing. Colloids Interface Sci. Commun..

[47] (1994). Textiles—Yarn from Packages—Determination of Linear Density (Mass per Unit Length) by the Skein Method.

[48] (2009). Textiles—Yarns from Packages—Determination of Single-End Breaking Force and Elongation at Break Using Constant Rate of Extension (CRE) tester (ISO 2062:2009).

[49] Twarowska-Schmidt K. (2012). Influence of drawing parameters on the properties of melt spun poly(lactic acid) fibres. Fibres Text. East. Eur..

[50] Phuphuak Y., Miao Y., Zinck P., Chirachanchai S. (2013). Balancing crystalline and amorphous domains in PLA through star-structured polylactides with dual plasticizer/nucleating agent functionality. Polymer.

[51] Liu S., Wu G., Chen X., Zhang X., Yu J., Liu M., Zhang Y., Wang P. (2019). Degradation behavior in vitro of carbon nanotubes (CNTs)/poly(lactic acid) (PLA) composite suture. Polymers.

[52] Kong Y., Hay J.N. (2002). The measurement of the crystallinity of polymers by DSC. Polymer.

[53] Krikštanavičienė K., Stanys S., Jonaitienė V. (2014). Dependence of Polypropylene Yarn Mechanical Properties on Manufacturing Parameters. Mater. Sci..

[54] (2017). Implants for Surgery—Homopolymers, Copolymers and Blends on Poly(lactide)—In Vitro Degradation Testing.

[55] Caronna F., Glimpel N., Paar G.P., Gries T., Blaeser A., Do K., Dolan E.B., Ronan W. (2022). Manufacturing, characterization, and degradation of a poly(lactic acid) warp-knitted spacer fabric scaffold as a candidate for tissue engineering applications. Biomater. Sci..

[56] Kim J.W., Park S., Sung Y.W., Song H.J., Yang S.W. (2023). Evaluation of antiviral compounds from Commiphora molmol myrrh Evaluation of Antiviral Compounds from Commiphora molmol Myrrh Resin and Their Promising Application with Biochar. Preprints.

[57] Batiha G.E.S., Wasef L., Teibo J.O., Shaheen H.M., Zakariya A.M., Akinfe O.A., Teibo T.K.A., Al-kuraishy H.M., Al-Garbee A.I., Alexiou A. (2023). Commiphora myrrh: A phytochemical and pharmacological update. Naunyn-Schmiedeberg’s Arch. Pharmacol..

[58] Tabur M.A. (2021). Characterization of Myrrh Essential Oil with GC-MS and Investigation Antibacterial Effects on *Salmonella* spp.. Süleyman Demirel Univ. Fac. Arts Sci. J. Sci..

[59] Hanuš L.O., Řezanka T., Dembitsky V.M. (2005). Myrrh–Commiphora Chemistry. Biomed. Pap..

[60] Yu W., Wang X., Ferraris E., Zhang J. (2019). Melt crystallization of PLA/Talc in fused filament fabrication. Mater. Des..

[61] Zhang H., Bai H., Wang N., Zhang Q., Fu Q. (2023). The study of correlations among the process condition, structure and property for poly(l-lactide) fibers. J. Eng. Fiber. Fabr..

[62] Gajjar C.R., Stallrich J.W., Pasquinelli M.A., King M.W. (2021). Process-Property Relationships for Melt-Spun Poly(lactic acid) Yarn. ACS Omega.

[63] Hossain M.T., Shahid M.A., Mahmud N., Habib A., Rana M.M., Khan S.A., Hossain M.D. (2024). Research and application of polypropylene: A review. Discov. Nano.

[64] Biomax® Thermal 300-DuPont-Datasheet. https://polymer-additives.specialchem.com/product/a-dupont-biomax-thermal-300.

[65] Alaburdaitė R., Krylova V. (2023). Polypropylene film surface modification for improving its hydrophilicity for innovative applications. Polym. Degrad. Stab..

[66] Tolinski M. (2015). Ultraviolet Light Protection and Stabilization. Additives for Polyolefins.

[67] Bones D.L., Henricksen D.K., Mang S.A., Gonsior M. (2010). Appearance of strong absorbers and fluorophores in limonene-O Appearance of strong absorbers and fluorophores in limonene-O 3 secondary organic aerosol due to NH + 4 -mediated chemical aging over long time scales. J. Geophys. Res. Atmos..

[68] Fu X., Zhang T., Zhang W., Zhong Y., Fang S., Wang G., Li Y., Deng Y., Liu X., Li H. (2023). Melt-blended PLA/curcumin-cross-linked polyurethane fi lm for enhanced UV-shielding ability. e-Polymers.

[69] Nanni A., Battegazzore D., Frache A., Messori M. (2019). Thermal and UV aging of polypropylene stabilized by wine seeds wastes and their extracts. Polym. Degrad. Stab..

[70] Ali A.M., El-Dessouky H.M. (2019). An insight on the process–property relationships of melt spun polylactic acid fibers. Text. Res. J..

[71] Yahyaoui M., Gordobil O., Herrera Díaz R., Abderrabba M., Labidi J. (2016). Development of novel antimicrobial films based on poly(lactic acid) and essential oils. React. Funct. Polym..

[72] Ferreira R.R., Farina M.C., Maia A., Torin R.F.S. (2023). PLA Films Containing Montmorillonite Nanoclay–Citronella Essential Oil Hybrids for Potential Active Film Formulation. Macromol.

[73] Biswal A.K., Vashisht I., Khan A., Sharma S., Saha S. (2019). Synthesis, characterization and antibacterial activity of thymol-loaded polylactic acid microparticles entrapped with essential oils of varying viscosity. J. Mater. Sci..

[74] Strasakova M., Pummerova M., Filatova K., Sedlarik V. (2021). Immobilization of Caraway Essential Oil in a Polypropylene Matrix for Antimicrobial Modification of a Polymeric Surface. Polymers.

[75] Qiao B., Teyssedre G., Laurent C. Field and electron beam-induced luminescence phenomena in polypropylene thin films. Proceedings of the IEEE 11th International Conference on the Properties and Applications of Dielectric Materials, 2015 (ICPADM).

[76] Marra A. (2017). Assessment on the Effects of ZnO and Coated ZnO Particles on iPP and PLA Properties for Application in Food Packaging. Coatings.

[77] Cuiffo M.A., Snyder J., Elliott A.M., Romero N., Kannan S., Halada G.P. (2017). Impact of the fused deposition (FDM) printing process on polylactic acid (PLA) chemistry and structure. Appl. Sci..

[78] Gopanna A., Mandapati R.N., Thomas S.P., Rajan K., Chavali M. (2019). Fourier transform infrared spectroscopy (FTIR), Raman spectroscopy and wide-angle X-ray scattering (WAXS) of polypropylene (PP)/cyclic olefin copolymer (COC) blends for qualitative and quantitative analysis. Polym. Bull..

[79] Johnson-restrepo B., Banquet-tera J., Fontalvo-gomez M., Roman R.J. (2016). Linear and Nonlinear Calibration Methods for Predicting Mechanical Properties of Polypropylene Pellets Using Raman Spectroscopy. Appl. Spectrosc..

[80] Brody R.H., Edwards H.G.M., Pollard A.M. (2002). Fourier Transform-Raman Spectroscopic Study of Natural Resins of Archaeological Interest. Biopolym. Orig. Res. Biomol..

[81] Puchowicz D., Cieslak M., Pathak C.S., Kumar S. (2021). Raman Spectroscopy in the Analysis of Textile Structures. Recent Developments in Atomic Force Microscopy and Raman Spectroscopy for Materials Characterization.

[82] Edwards H.G.M., Falk M.J. (1997). Fourier-transform Raman spectroscopic study of frankincense and myrrh. Spectrochim. Acta-Part A Mol. Biomol. Spectrosc..

[83] Andreassen E. (1999). Infrared and Raman Spectroscopy of Polypropylene.

[84] Kuck K., Unterholzner A., Lipowicz B., Schwindl S., Jürgenliemk G., Schmidt T.J., Heilmann J. (2023). Terpenoids from Myrrh and Their Cytotoxic Activity against HeLa Cells. Molecules.

[85] Yu W., Liu Y., Wang L., Shi J. (2019). Cu Nanoparticle-Modified High-Density Polyethylene Monofilament and Its Antifouling Performance on Fishing Netting. Int. J. Polym. Sci..

[86] Alshehri M.A., Baskaradoss J.K., Geevarghese A., Ramakrishnaiah R., Tatakis D.N. (2015). Effects of myrrh on the strength of suture materials: An in vitro study. Dent. Mater. J..

[87] Eshaghi R., Mohsenzadeh M., Ayala-Zavala J.F. (2024). Bio-nanocomposite active packaging films based on carboxymethyl cellulose, myrrh gum, TiO_2_ nanoparticles and dill essential oil for preserving fresh-fish (*Cyprinus carpio*) meat quality. Int. J. Biol. Macromol..

